# Finite element biomechanical analysis of 3D printed intervertebral fusion cage in osteoporotic population

**DOI:** 10.1186/s12891-024-07221-7

**Published:** 2024-02-12

**Authors:** Jincheng Wu, Jun Miao, Guangdong Chen, Hanpeng Xu, Wangqiang Wen, Haoxiang Xu, Lizhu Liu

**Affiliations:** 1 Department of Emergency Trauma Surgery, The Second Affiliated Hospital of Hainan Medical University, Haikou City, Hainan China 48 Baishuitang Road, Longhua District, 571700; 2 Tianjin Hospital, Tianjin University, Tianjin, China; 3 Huazhong University of Science and Technology, Wuhan, Hubei China; 4 The First Affiliated Hospital of Hainan Medical University, Haikou City, Hainan China; 5 The Second People’s Hospital of Hefei, Hefei, Anhui China

**Keywords:** Interbody fusion, Osteoporosis, Lumbar spine, Finite element model, 3D printed cage, Instrumentation failure

## Abstract

**Objective:**

To study the biomechanical characteristics of each tissue structure when using different 3D printing Cage in osteoporotic patients undergoing interbody fusion.

**Methods:**

A finite element model of the lumbar spine was reconstructed and validated with regarding a range of motion and intervertebral disc pressure from previous in vitro studies. Cage and pedicle screws were implanted and part of the lamina, spinous process, and facet joints were removed in the L4/5 segment of the validated mode to simulate interbody fusion. A 280 N follower load and 7.5 N·m moment were applied to different postoperative models and intact osteoporotic model to simulate lumbar motion. The biomechanical characteristics of different models were evaluated by calculating and analyzing the range of motion of the fixed and cephalic adjacent segment, the stress of the screw-rod system, the stress at the interface between cage and L5 endplate, and intervertebral disc pressure of the adjacent segment.

**Results:**

After rigid fixation, the range of motion of the fixed segment of model A-C decreased significantly, which was much smaller than that of the osteoporotic model. And with the increase of the axial area of the interbody fusion cages, the fixed segment of model A-C tended to be more stable. The range of motion and intradiscal pressure of the spinal models with different interbody fusion cages were higher than those of the complete osteoporosis model, but there was no significant difference between the postoperative models. On the other hand, the L5 upper endplate stress and screw-rod system stress of model A-C show a decreasing trend in different directions of motion. The stress of the endplate is the highest during flexion, which can reach 40.5 MPa (model A). The difference in endplate stress between models A-C was the largest during lateral bending. The endplate stress of models A and B was 150.5% and 140.9% of that of model C, respectively. The stress of the screw-rod system was the highest during lateral bending (model A, 102.0 MPa), which was 108.4%, 102.4%, 110.4%, 114.2% of model B and 158.5%, 110.1%, 115.8%, 125.4% of model C in flexion, extension, lateral bending, and rotation, respectively.

**Conclusions:**

For people with osteoporosis, no matter what type of cage is used, good immediate stability can be achieved after surgery. Larger cage sizes provide better fixation without significantly increasing ROM and IDP in adjacent segments, which may contribute to the development of ASD. In addition, larger cage sizes can disperse endplate stress and reduce stress concentration, which is of positive significance in preventing cage subsidence after operation. The cage and screw rod system establish a stress conduction pathway on the spine, and a larger cage greatly enhances the stress-bearing capacity of the front column, which can better distribute the stress of the posterior spine structure and the stress borne by the posterior screw rod system, reduce the stress concentration phenomenon of the nail rod system, and avoid exceeding the yield strength of the material, resulting in the risk of future instrument failure.

## Introduction

Global Burden of Diseases, Injuries, and Risk Factors Study has assessed hundreds of diseases in recent decades and found that low back pain is one of the main reasons that seriously affects our quality of life, causing a huge burden on society and medical care [1]. Therefore, the study of the etiology of low back pain has been paid more attention. Due to the complexity of the lumbar musculoskeletal system, there are various causes of low back pain. With the progress of medical treatment, people pay more attention to the psychological aspect. Based on much research on the etiology of low back pain, the concept of biopsychosocial pain syndrome was proposed. The biological factors include abnormal shape and function of the spine. Such as spinal tumors, spinal fractures, infections, disc degeneration, and so on [2–5].

For low back pain, we generally choose conservative treatment, and only when conservative treatment fails and there are serious neurological symptoms, surgical treatment is considered [6]. Among the surgical treatments for low back pain, interbody fusion plays an important role. The purpose of surgery is achieved by restoring the physiological curvature of the spine, decompressing nerves, and using various cages to stabilize degenerated segments for interbody fusion [7, 8]. Many patients benefit from this, and the symptoms of low back pain are significantly relieved. However, interbody fusion can also lead to related complications such as adjacent segment degeneration and cage subsidence [4, 8]. Studies have reported cage subsidence rates of 15.9–70% and failure of bone fusion rates of 5%-35%, with even higher rates in patients with multiple spinal motor segments [9, 10]. At present, the number of people undergoing interbody fusion has increased greatly, and the high postoperative complications have been paid more and more attention because they can seriously affect people's quality of life. Cage subsidence and failure of bone fusion mean changes in the physiological curvature of the spine, decreased intervertebral height, and even compression of the nerve resulting in recurrent lower back pain [10, 11]. Therefore, how to reduce cage subsidence, promote bone fusion, and reduce postoperative complications of interbody fusion has become the focus of research. Recent studies on the factors influencing cage subsidence have shown that different surgical approaches, endplate integrity, internal fixation systems, and cage characteristics all affect the probability of cage subsidence [10–13].

Osteoporosis is one of the common diseases in the elderly. With the progress of population aging, the number of osteoporosis patients undergoing interbody fusion is further increasing. Some studies have analyzed and reported the risk factors related to cage subsidence, among which BMD was found to be highly correlated with cage subsidence [10–12]. BMD is an important determinant of fracture risk in the elderly [14]. Low BMD reduces the failure load strength of the endplate surface, resulting in the weakening of endplate load transfer strength, which increases the risk of cage subsidence [15]. Previous studies on the factors affecting the rate of interbody fusion have found that the BMD of patients with “union” was significantly higher than those with “non-union” and “undetermined-union” [13]. Therefore, surgeons have been vigilant to prevent the occurrence of complications after interbody fusion in osteoporotic population. The surgical techniques, selection of bone graft materials and cage have been the focus of people's research. For example, Jain et al. investigated the biomechanics of different size pedicle screws in osteoporosis models[16]. According to the latest report, the material of cage does not affect bone fusion. and there is no significant difference in the fusion rate between cages made of PEEK, titanium, tantalum and other materials. The ideal biological agents with osteoinductive, osteoconductive and osteogenic properties can improve the success rate of bone fusion [13, 17]. Although some in vitro experiments have studied the effect of changes in BMD on the destructive strength of the vertebral endplate, and the correlation between different cage designs and cage subsidence [18–21]. However, due to the limitations of materials, it is impossible to simulate the biomechanical properties of vertebrae with different bone mineral density, which has some limitations. However, in some clinical case studies, there are some factors that may lead to bias in analysis results, such as small sample size, different BMD of patients, and differences in surgical procedures, and the conclusions are controversial. At present, there is a lack of biomechanical study that can more accurately reflect the effect of bone-cage-bone interface area on cage subsidence and other structures of the spine.

Unlike in vitro experiments, finite element analysis applications computers can build the required biomechanical models according to people's design needs. Finite element analysis can more intuitively reflect the mechanical results of each part of the model. Experimental parameters can be modified to avoid result errors caused by individual differences in vitro tests and improve the accuracy of calculation [22]. Finite element analysis has been applied to the field of bone biomechanics since 1972, and with the development of computers, finite element analysis has been applied more and more widely in the medical field [23–25]. Finite element analysis can build models based on imaging data, accurately restore the spinal structure, and can be endowed with different material properties to simulate muscles, ligaments, intervertebral discs with different grades of degeneration, and other structures, so as to better simulate the real movement of the human spine. It can predict possible behavioral consequences and complications in the medical field by constructing a good model, which is corroborated by in vitro trials. The results of finite element analysis are reproducible, which simplifies some research limitations of in vitro experiments and is more convenient in experimental conditions [26, 27].

With the development of 3D printing technology, the personalized design of 3D printing cage can ensure a close fit with the vertebral body, and its microporous structure is conducive to bone fusion [28]. The musculoskeletal system of different individuals is diverse, and 3D printing technology can be customized according to its technical advantages, which makes it develop rapidly in the medical field. 3D printing technology is combined with bioengineering and other disciplines so that the product has good histocompatibility, osteogenic properties, bone induction, and good bone conduction. In order to study the biomechanical characteristics of cage with different coaxial areas during interbody fusion in osteoporotic population, observe the stress and strain distribution of endplate, and evaluate its role in preventing postoperative complications in osteoporotic population, the finite element method was used to create a finite element model of human lumbar spine and simulate the surgical process. In spinal interbody fusion, the 3D-printed cage's personalized design ensures a tight fit to the vertebral body, and its microporous structure provides better bone inductance for bone fusion [28]. The mechanical characteristics of various structures and instruments of the lumbar spine were analyzed and calculated after surgery to provide a theoretical basis for surgeons to choose cage when performing interbody fusion in osteoporosis patients, so as to improve the prognosis of patients and reduce the possible complications.

## Materials and methods

### Intact FE model

Data of the L1-5 lumbar spine FE model were collected from a healthy adult male volunteer (24 years old, weight 67 kg, height 173 cm). The volunteer had no previous history of trauma or fracture. Any spinal diseases were excluded by clinical imaging examination to establish a normal intact FE model. The volunteers were recruited by the Spinal surgery Department of Tianjin Hospital, and the subjects were informed in detail of the risks and protective measures that might be faced in the experimental steps, such as the items and times of X-ray or CT examinations were in line with the commonly used clinical radiological standards, and the radiation dose was small, but usually did not have adverse effects. The volunteer signed informed consent forms in accordance with the relevant regulations, which were submitted it to the Ethics Committee of Tianjin Hospital for approval. All clinical investigations were conducted in strict accordance with the principles of the Declaration of Helsinki [29].

A 64-slice spiral computed tomography scanner (Siemens, Erlangen, Germany) was used to obtain tomographic data of L1-5 vertebrae in DICOM format with a 0.625 mm interslice interval. The image data were imported into mimics 20.0 (Materialise Inc., Leuven, Belgium) to reconstruct a 3D surface model of L1-5 vertebrae and saved it in STL format. The modeling method referred to previous researches [30, 31]. The model was imported into 3-Matic 12.0 software (Materialise Inc.) to perform wrapping, smoothing and Boolean operation. The redundant triangular surfaces were removed to generate more detailed 3D images, and the structures of facet joints, intervertebral discs and nucleus pulposus were initially constructed [32]. Then the lumbar model was imported into GeomagicStudio12.0 (Geomagic Inc., USA), and the solid model was constructed by using the functions of smoothing, accurate surface, editing contour, constructing surface patch, constructing grille, and so on, and exported in iges format [29]. After that, the more accurate model was imported into Hypermesh2017 (Altair Engineering, Troy, Michigan, USA) for mesh division and construction of seven ligament (ALL: anterior longitudinal ligament; PLL: posterior longitudinal ligament; LF: ligamentum flavum; CL: capsular ligament; ISL: interspinous ligament; SSL: supraspinous ligament; ITL: Intertransverse ligament). Finally, the model was imported into Abaqus 2019 (Simulia, Johnston, RI, USA) for FE analysis [29, 33, 34].

**Fig. 1:**
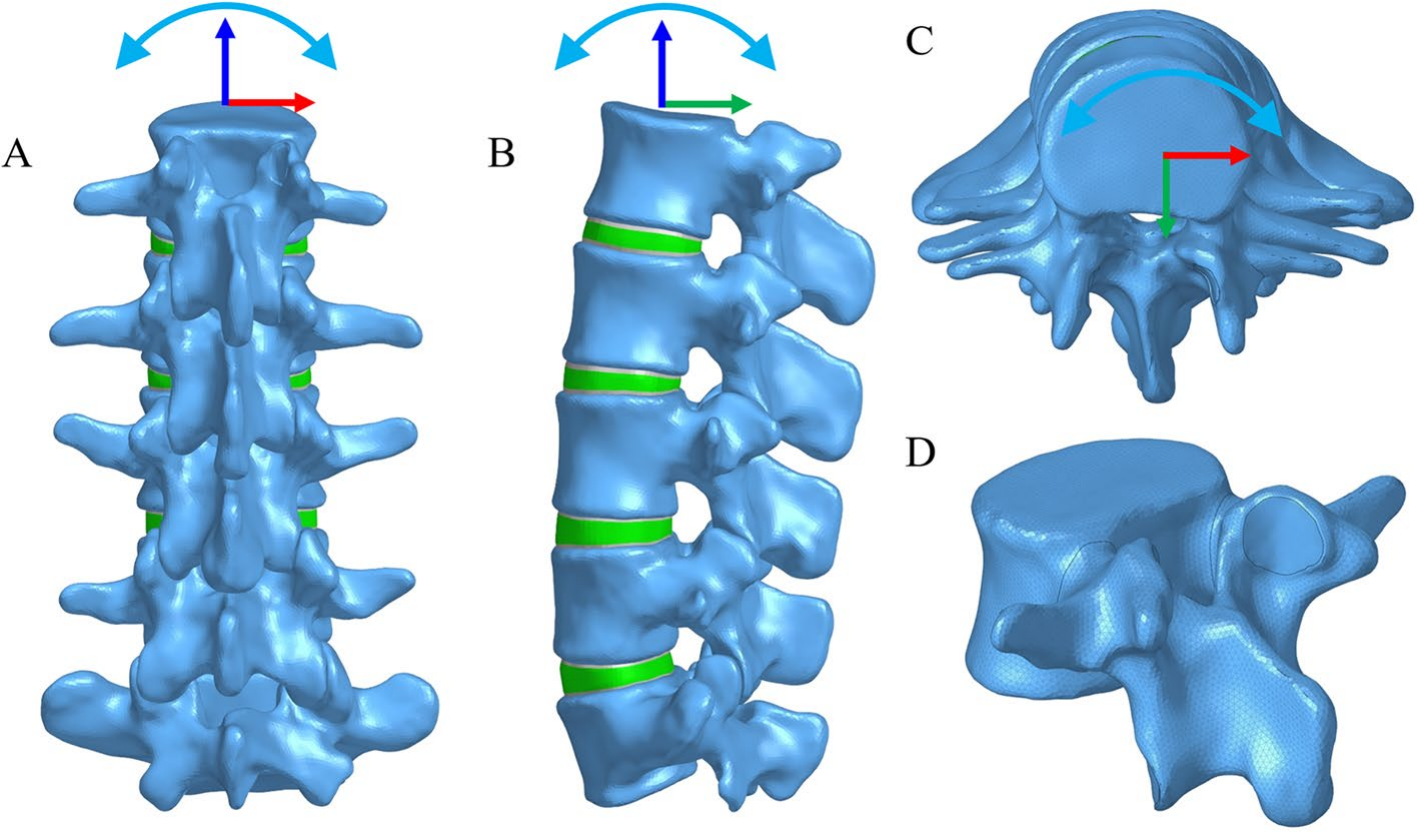
Finite element model of lumbar spine (L1-5) and schematic diagram of motion direction. **A** Posterior view. **B** Lateral view. **C** Top view. **D** Vertebral meshed image

As shown in Fig. 1, a three-dimensional FE model of L1-5 lumbar spine was reconstructed.

**Fig. 2:**
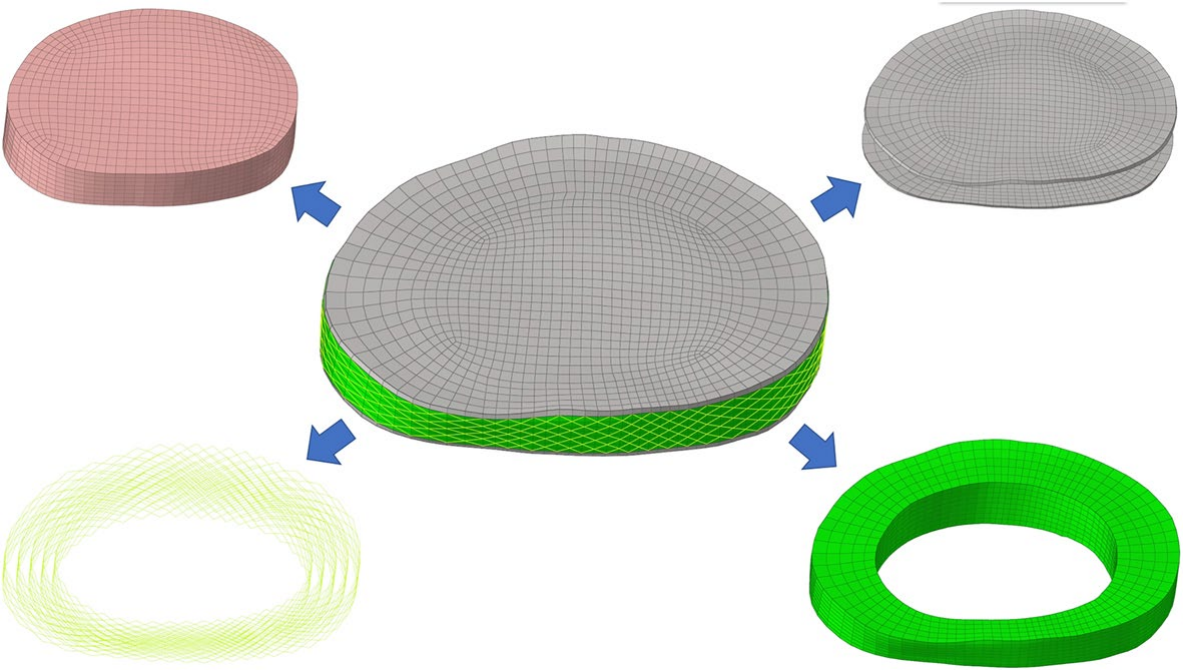
Schematic diagram of the structure of each part of the intact intervertebral disc

Models with mesh sizes of 1, 1.5 and 2 mm were designed, and the maximum VMS stress of the model was compared with that of the 0.5 mm model, respectively. When the error was between 5%, the model was considered to be convergent [35]. Considering the computational cost and the accuracy of the results, we chose the 1 mm mesh size. The whole lumbar model consists of 1,483,153 elements and 364,027 nodes. The vertebral body was composed of cortical, cancellous and posterior bone structures, which were divided by tetrahedral mesh. The thickness of cortical bone and articular cartilage was 1 mm and 0.2 mm respectively [30, 36]. The intervertebral disc uses hexahedral mesh, which is composed of annulus ground substance, nucleus pulposus, annulus fibers and cartilaginous endplate (Fig. 2). The thickness of the upper and lower endplate is 0.5 mm [36], and the nucleus pulposus accounts for 30%-40% of the total disc [37–39]. Ligaments and annulus fibers were simulated by using tension-only truss elements [34], in which fibers were constructed from inside to outside and embedded into the annulus ground substance at an angle of ± 30°. The elastic strength increased proportionally from the innermost (360 MPa) to the outermost fibers (550 MPa) [37, 40, 41]. To simulate the mechanical properties of the osteoporotic population model, we referred to previous research results and found that osteoporosis would reduce the Young's modulus of all bone structures while other structural properties remained unchanged. Based on the measurement of BMD in healthy and osteoporotic people, Young's modulus of cortical bone, posterior bone and endplate in osteoporotic people decreased by 33% and cancellous bone by 66%, which would lead to a 35% reduction in vertebral compression stiffness [42, 43]. The specific material properties of each structure were described in Tables 1 and 2 [44, 45].

**Table 1:** Material properties used in finite element model

Component	Young’s Modulus (MPa)	Poisson Ratio	Cross-Sectional Area(mm^2^)
Vertebra			
Cortical bone	12,000	0.3	-
Cancellous bone	100	0.2	-
Posterior element	3500	0.25	-
Sacrum	5000	0.2	-
Facet	11	0.2	-
Disc			
Endplate	24	0.4	-
Nucleus pulpous	1	0.49	-
Annulus ground substance	2	0.45	-
Annulus fibers	360–550	-	0.15
Implants			
Cage (titanium alloy)	110,000	0.3	-
Screws and rods (titanium alloy)	110,000	0.3	-
Bone graft	100	0.2	-
Porous part of cage	675	0.3	-
Ligaments			
ALL	7.8	-	63.7
PLL	10	-	20
LF	15	-	40
CL	7.5	-	30
ISL	10	-	40
SSL	8	-	30
ITL	10	-	1.8

**Table 2:** Material properties of bone structure in normal and mild osteoporosis

	Cortical bone	Cancellous bone	Posterior element	Endplate
Normal	12,000 MPa	100 MPa	3500 MPa	24 MPa
Mild osteoporosis	8040 MPa	34 MPa	2345 MPa	16.08 MPa

### Model simulation

**Fig. 3:**
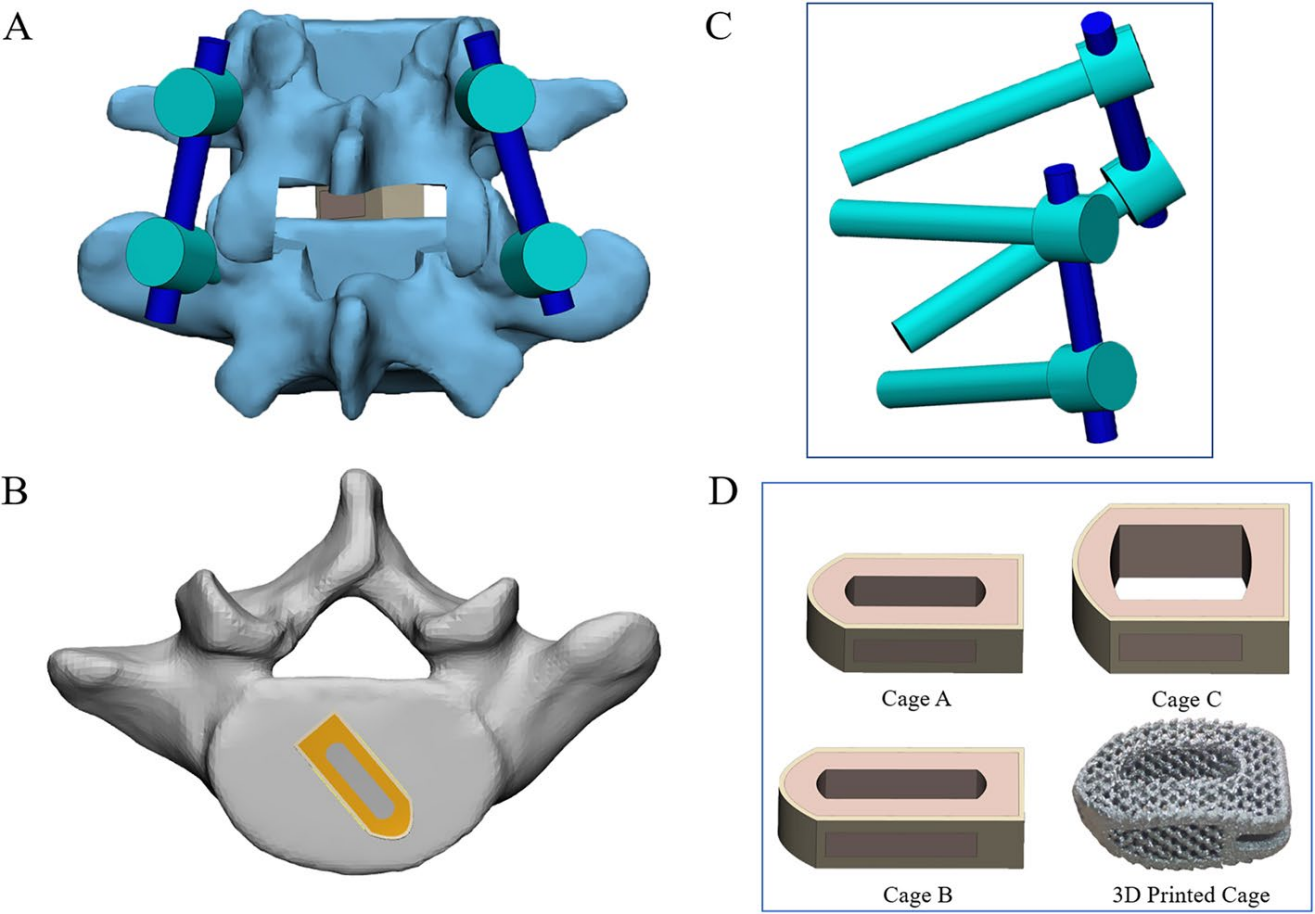
**A** Schematic diagram of operation segment. **B** Cage and vertebral body position relationship diagram. **C** Simplified screw rod model. **D** 3D printed cage and different axial areas of cage model

Lumbar degenerative diseases often occur in the L4/5 segment, so it was selected for posterior lumbar interbody fusion (PLIF) in this study. According to the usual resection range during PLIF, we removed the posterior part of spinous process and lamina of L4 vertebral body, the medial side of bilateral facet joint process of the L4/5 segment, the corresponding SSL, ISL, LF and CL, and the intervertebral disc of L4/5, and implanted internal fixation instruments at the same time (Fig. 3) [29].

In this experiment, the internal fixation instrumentation was constructed by Pro/Engineer5.0 software. In order to obtain a convenient internal fixation model, combined with previous research protocol, a simplified lumbar pedicle screw (diameter 6.5 mm, length 45 mm) and connecting rod (diameter 5.5 mm) were designed. Because this study does not consider the relative sliding of the screw and the vertebral body, in order to reduce the calculation cost on the premise of obtaining accurate results, we simplified the screw thread and replaced it with a long cylinder [46, 47]. To obtain rigid fixation in L4/5 segments, we placed a cage in the intervertebral space and two pedicle screws in L4 and L5 vertebrae respectively. The insertion point of pedicle screw is the intersection of the vertical line of the outer edge of the articular process and the horizontal line of the midpoint of the transverse process. The same tie contact was used at the screw-rod junctions and between the screw and the vertebral body to form a rigid connection and limit the relative movement [48–50].

Cage A (9 mm × 10 mm × 23 mm) is a commonly used cage type during PLIF. In order to more comprehensively analyze the effect of different axial area of cage on osteoporosis patients, two different sizes of titanium alloy cages were designed in this study (cage B: 9 × 10 × 26 mm, cage C: 15 × 10 × 23 mm). In order to distinguish them, we named the postoperative models with different sizes of cage as model A, B and C respectively. To achieve interbody union under internal fixation, we filled all cage graft holes with cancellous bone. We applied Boolean operation to remove the part of the cage in contact with the vertebral body and the geometric contact between the cage and the vertebral body was satisfied. Referring to the previous experimental method, the porous part of cage was simulated by a solid with smaller Young's modulus [51].

### FE model validation

The rationality of the intact model was verified by referring to the previous research methods of Renner et al. [52]. As with previous research methods, we fixed the bottom of the sacrum, limiting its displacement and rotation in all directions. Four pure moments (flexion: 8N·m, extension 6N·m, lateral bending ± 6N·m, rotation ± 4N·m) were applied to the centre of the upper surface of L1 to simulate the motion of lumbar spine. We defined the motion of the sagittal, coronal and transverse planes as flexion and extension, lateral bending and rotation, respectively. The range of motion (ROM) of each segment was compared with the results of previous studies. In addition to verifying the ROM of each segment of the lumbar model, we also verified the intervertebral disc pressure (IDP) of the L4/5 segment. Referring to previous studies by Brinckmann et al., the IDP of L4/5 segment was measured by applying a gradually increasing compression force (300N, 1000N) [53, 54].

### Boundary and loading conditions

Each model component was imported into ABAQUS software in INP format for final model analysis and calculation. We applied the same conditions to all models to constrain the base of the sacrum and restrict its movement in all directions. An axial load of 280N was applied to the L1 vertebral body to simulate part of the human body weight borne by the lumbar spine [36, 49]. A moment of 7.5N·m was applied to simulate the flexion, extension, lateral bending and rotation of the lumbar spine.

### Assessment indexes

In this study, the biomechanical characteristics between different models were compared by calculating and measuring the ROM of the fusion and cephalic adjacent segment, the stress of screw-rod system, IDP of the adjacent segment, and the stress at the interface between cage and L5 endplate. To evaluate the effect of different axial areas of cage on osteoporosis patients undergoing interbody fusion, and to provide theoretical basis for surgeons.

## Results

### FE model validation

**Fig. 4:**
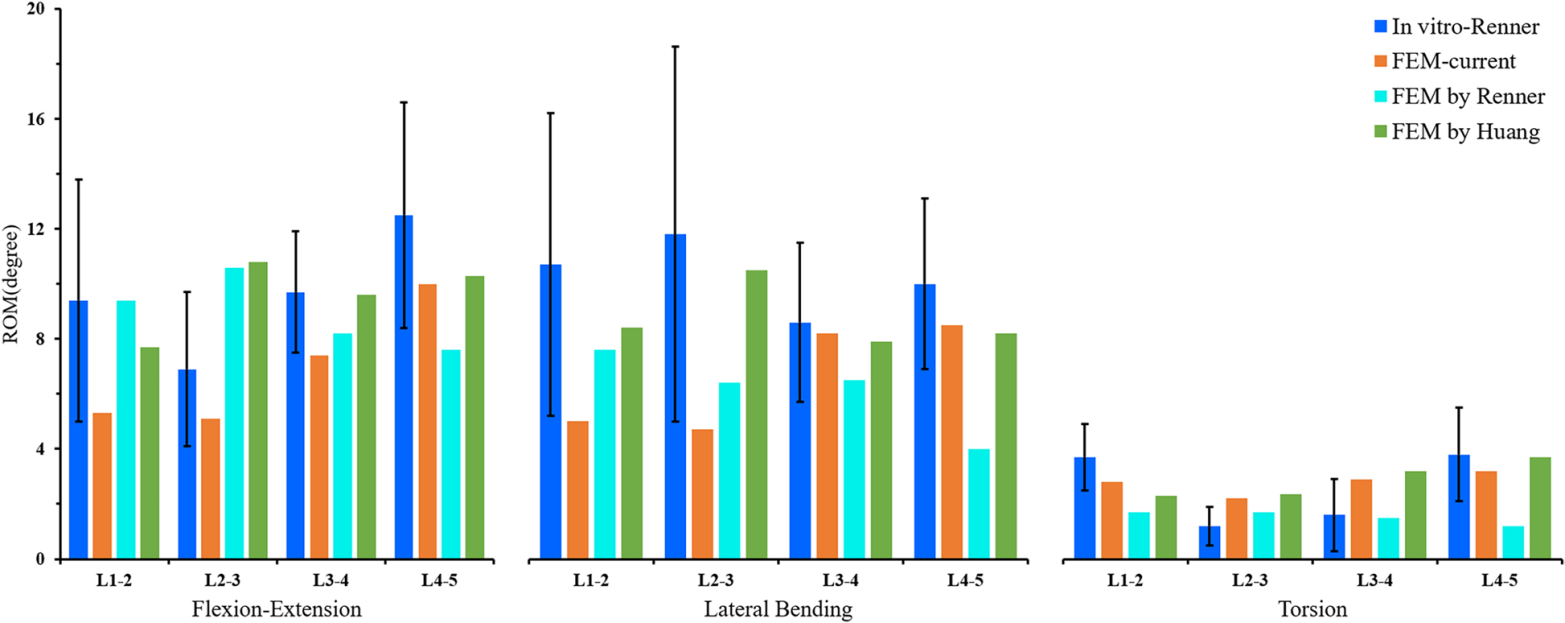
Comparison of the ROM of each motion segment between the current and previous studies

**Fig. 5:**
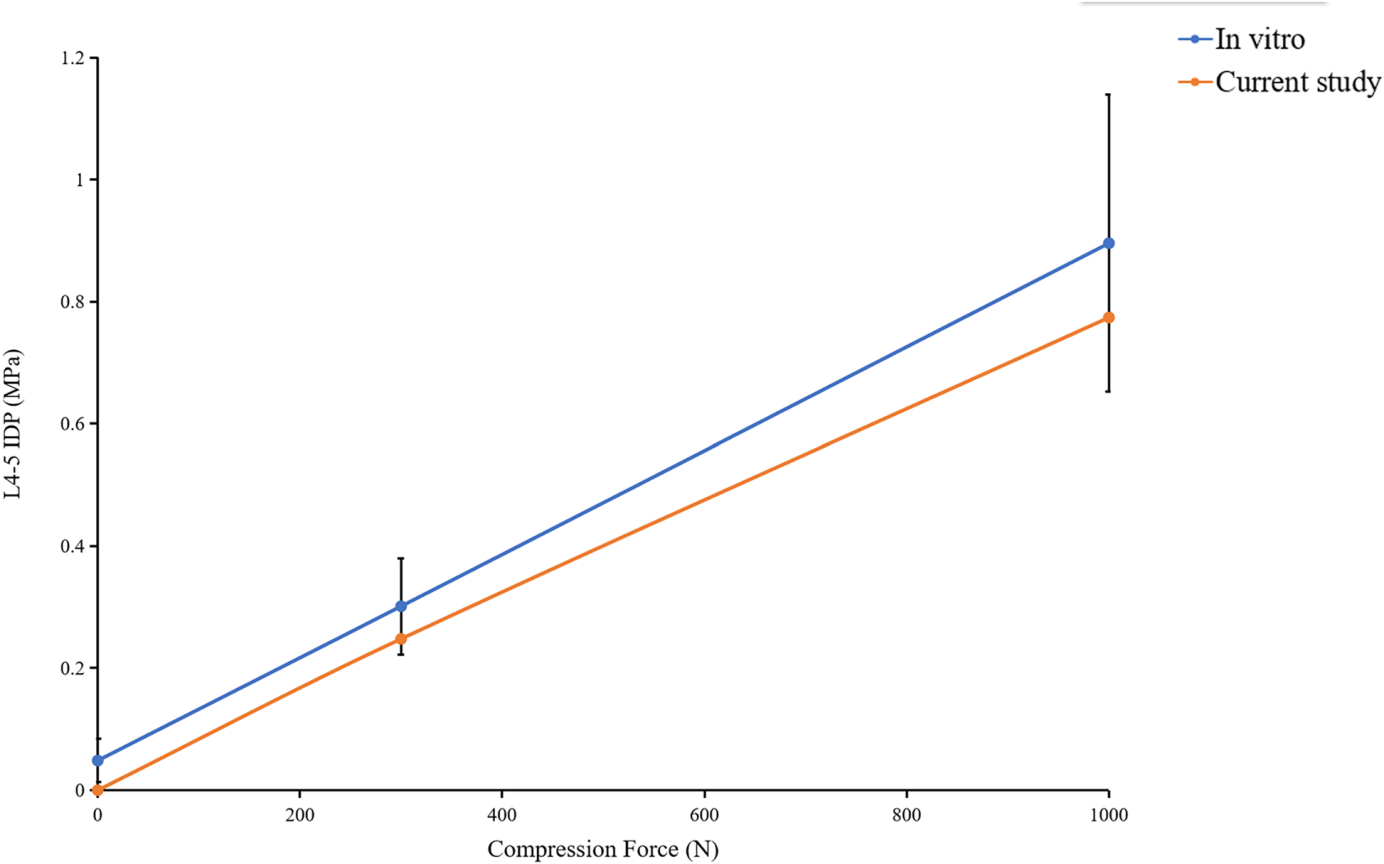
Comparison of the IDP of L4/5 under different compression loads between the current and previously studies

In this study, the rationality of the lumbar spine model was verified by comparing the ROM of each lumbar segment and the IDP of L4/5 segment with the previous study. The results showed that the ROM of each segment was in good agreement with the previous in vitro experiments of Renner et al. [52] and the FE study of Huang et al. (Fig. 4 and Table 3) [45]. The L4/5 IDP in this study also showed the same increasing trend as Brinckmann et al. [54] and Sengul et al. [48] under gradually increasing compression loads (Fig. 5). The results showed that the FE model of this study was consistent with the physiological characteristics of the human body, and was effective for our next research.

**Table 3:** The Rom of each segment in current and previous studies

		in vitro-Renner	FEM-current	FEM by Renner	FEM by Huang
Flexion–Extension (degree)	L1/2	9.4 ± 4.4	5.3	9.4	7.7
	L2/3	6.9 ± 2.8	5.1	10.6	10.8
	L3/4	9.7 ± 2.2	7.4	8.2	9.6
	L4/5	12.5 ± 4.1	10	7.6	10.3
Lateral Bending (degree)	L1/2	10.7 ± 5.5	5	7.6	8.4
	L2/3	11.8 ± 6.8	4.7	6.4	10.5
	L3/4	8.6 ± 2.9	8.2	6.5	7.9
	L4/5	10 ± 3.1	8.5	4	8.2
Torsion (degree)	L1/2	3.7 ± 1.2	2.8	1.7	2.3
	L2/3	1.2 ± 0.7	2.2	1.7	2.35
	L3/4	1.6 ± 1.3	2.9	1.5	3.2
	L4/5	3.8 ± 1.7	3.2	1.2	3.7

### The ROM of the fixed segment

**Fig. 6:**
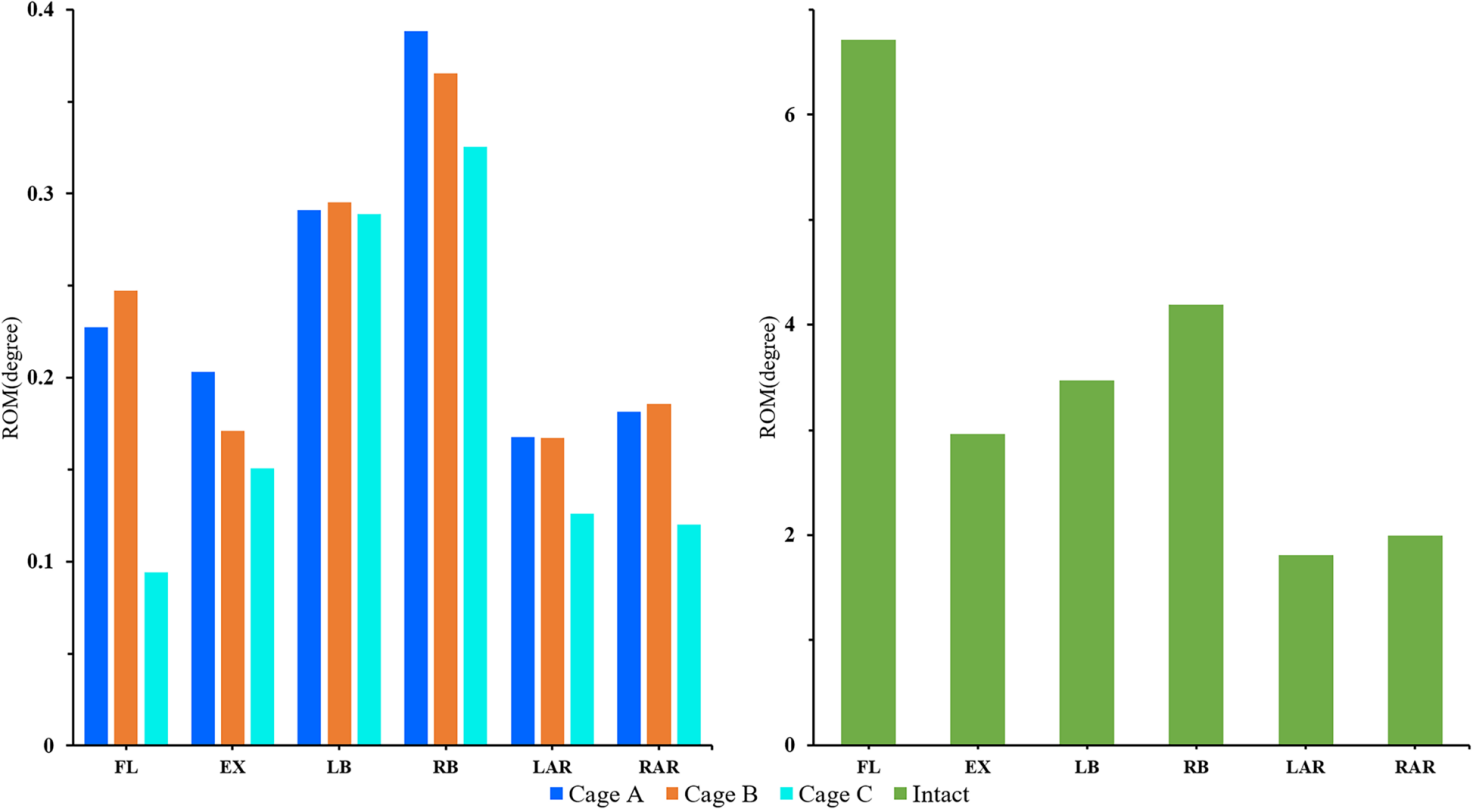
Comparison of the ROM between the intact osteoporotic model and postoperative models at the fusion segment (L4/5)

The results showed that the internal fixation device provided good stability in the fusion segment, and the ROM of postoperative models in all directions was obviously limited (Fig. 6). Postoperative models showed the best stability during flexion (98.6%). Even though the internal fixation device had the least limitation in lateral bending of postoperative models, it was 91.2% lower than that of the intact osteoporosis model. Among postoperative models, model C showed the smallest ROM in all directions, especially in flexion. Model A was similar to model B in rotation and left bending, but the ROM of model A was larger than model B in extension (118.7%) and right bending (106.3%).

### ROM of the cephalic adjacent segment

**Fig. 7:**
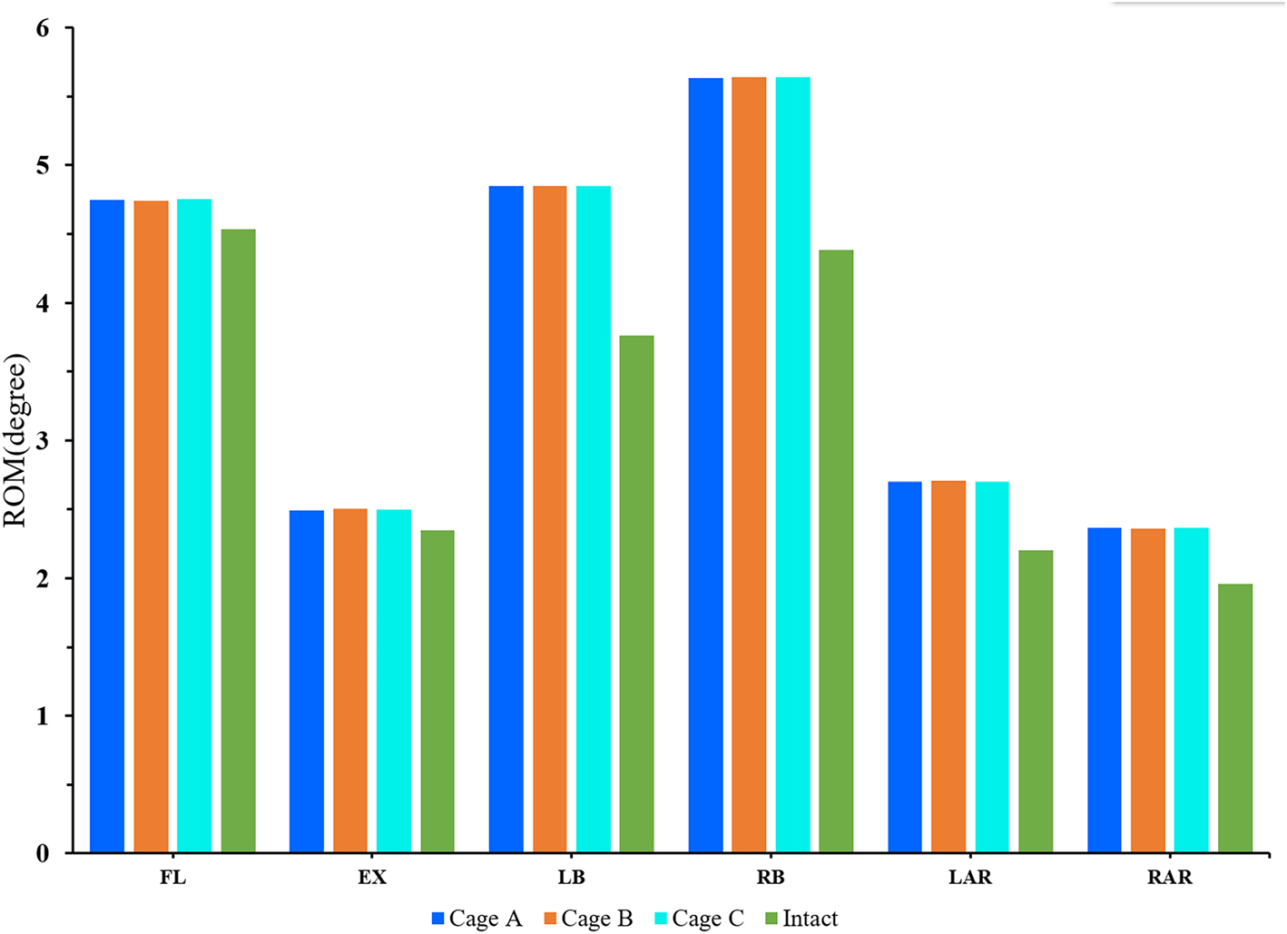
Comparison of the ROM between the different FE models at the cephalic adjacent segment (L3/4)

The ROM and trend of motion of each model in the cephalic adjacent segment were shown in Fig. 7. After cage and pedicle screw fusion at the L4/5 level, the ROM in the proximal cephalic segment was greater in all postoperative models than in osteoporosis models. The ROM of the adjacent segment in the postoperative lateral bending of the model was the largest, up to 5.6 degrees, and was the most different from that of the osteoporosis model, with an increase of 1.3 degrees (28.7%). Although the ROM of the postoperative model was larger than the direction of extension and rotation during flexion, the difference between the postoperative model and the osteoporosis model was the smallest, only 0.2 degree (4.9%). Although the ROM of postoperative model was increased after rigid internal fixation, the ROM of different postoperative models was similar in all directions, and the difference of ROM between different postoperative models was less than 0.1 degree.

### IDP of the adjacent segment

**Fig. 8:**
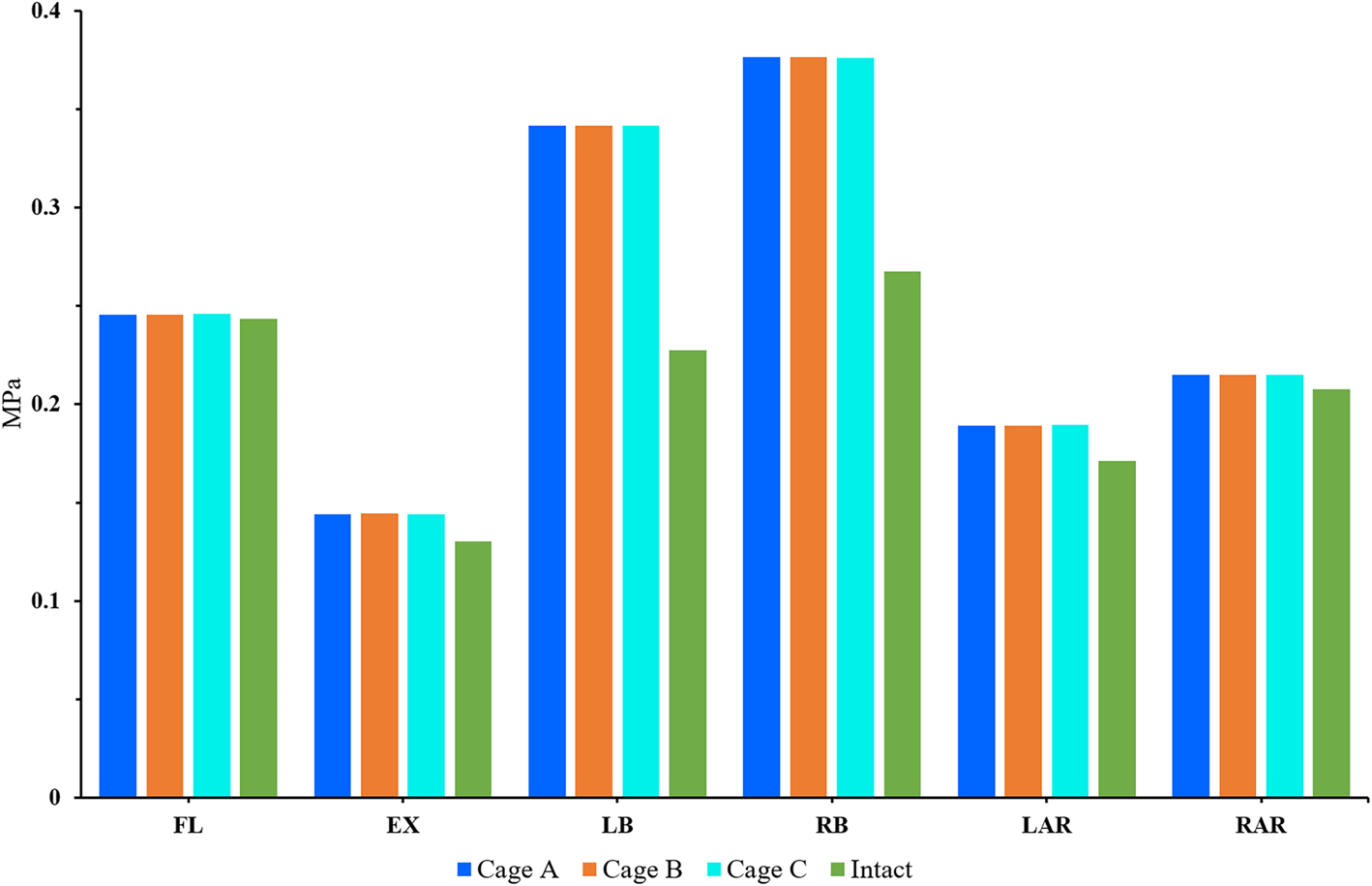
Comparison of the IDP between the intact osteoporotic model and postoperative models at the cephalic adjacent segment (L3/4)

The results showed that the IDP of the cephalic adjacent segment of postoperative models was higher than that of the intact osteoporosis model after L4/5 segment fixation (Fig. 8). Among them, the difference was the largest in lateral bending (44.9%) and the smallest in flexion (1.1%). Although the IDP in the three planes of motion of the postoperative model was higher than that in the intact osteoporosis model, the IDP was similar between them. Although there was no significant difference between the postoperative models, their IDP was significantly different in different directions. The maximum pressure is 0.3764 MPa when bending, followed by flexion (0.2459 MPa), rotation (0.2148 MPa) and extension (0.1444 MPa).

### Stress of the screw-rod system

**Fig. 9:**
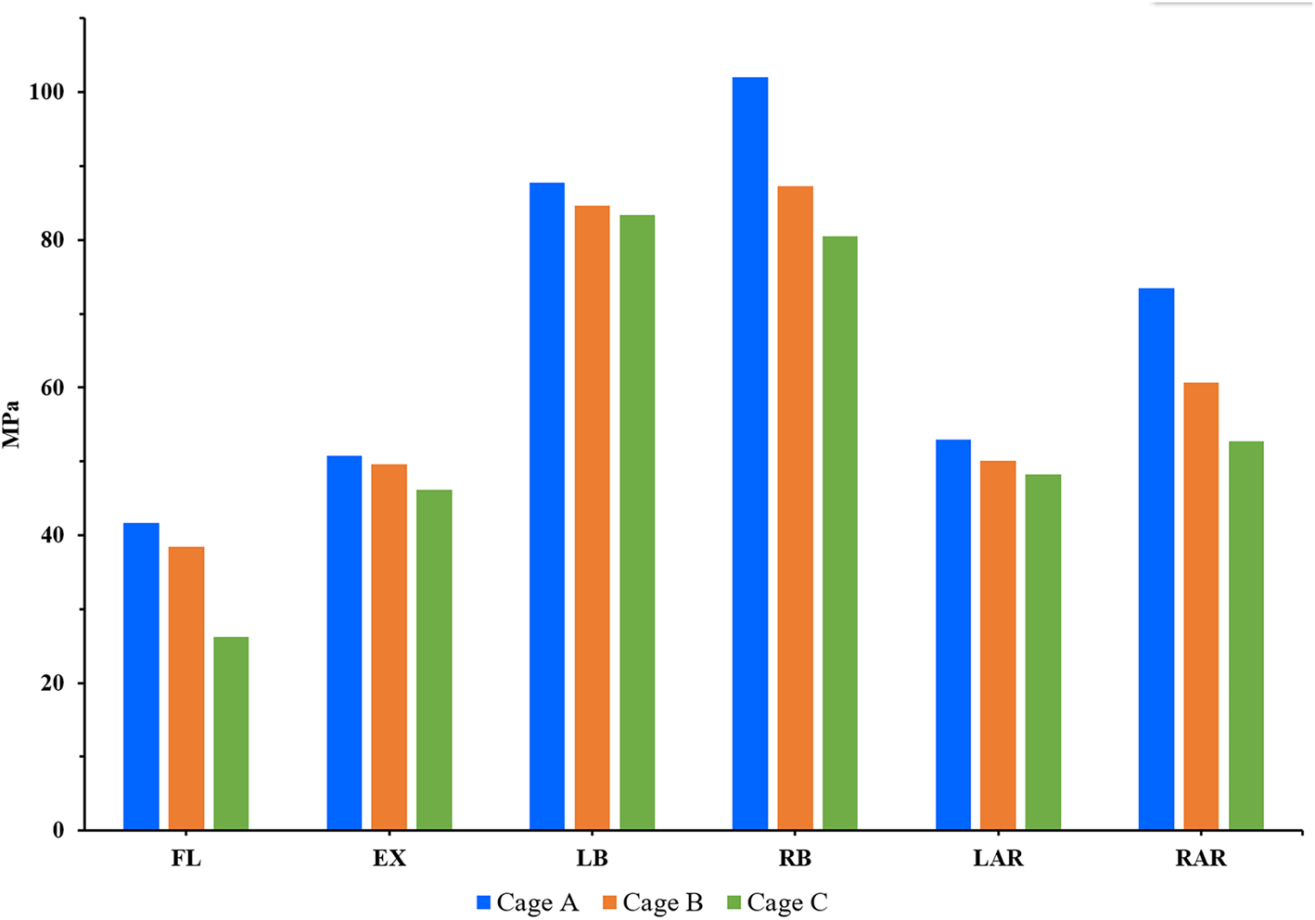
Comparison of the screw rod stress between the different postoperative models

**Fig. 10:**
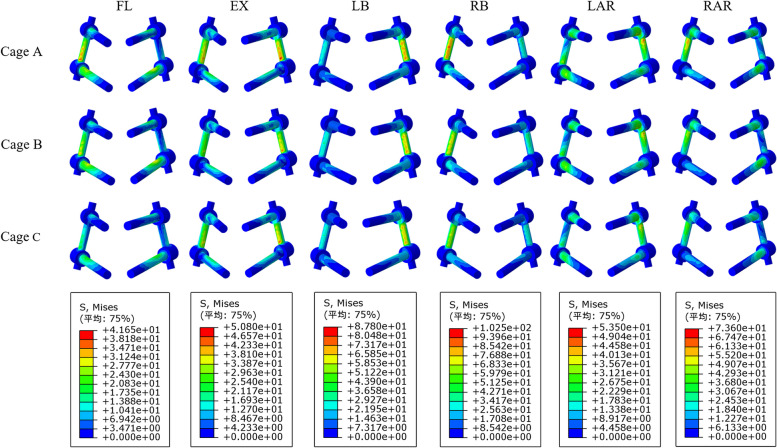
The stress distribution of the screw rod for postoperative model under each activity

The risk of screw fracture and loosening can be assessed by the stress distribution of the internal fixation system. The results showed that the screw rod stress of postoperative models has a good trend in all directions (Fig. 9). Regardless of the direction of motion, the screw rod stress of model A was always the largest, followed by model B, and model C was the smallest. Model A was 108.4%, 102.4%, 110.4%, 114.2% of model B and 158.5%, 110.1%, 115.8%, 125.4% of model C in flexion, extension, lateral bending, and rotational, respectively. The screw rod stress of postoperative models was the highest during the coronal movement, and the stress of model A was as high as 102.0 MPa in right bending. However, the screw rod stress of postoperative models was the lowest in sagittal movement, and the stress of model C was only 26.3 MPa in flexion. The stress magnitude and distribution range of the screw-rod system of the model are shown in Fig. 10.

### Stress of endplate

**Fig. 11:**
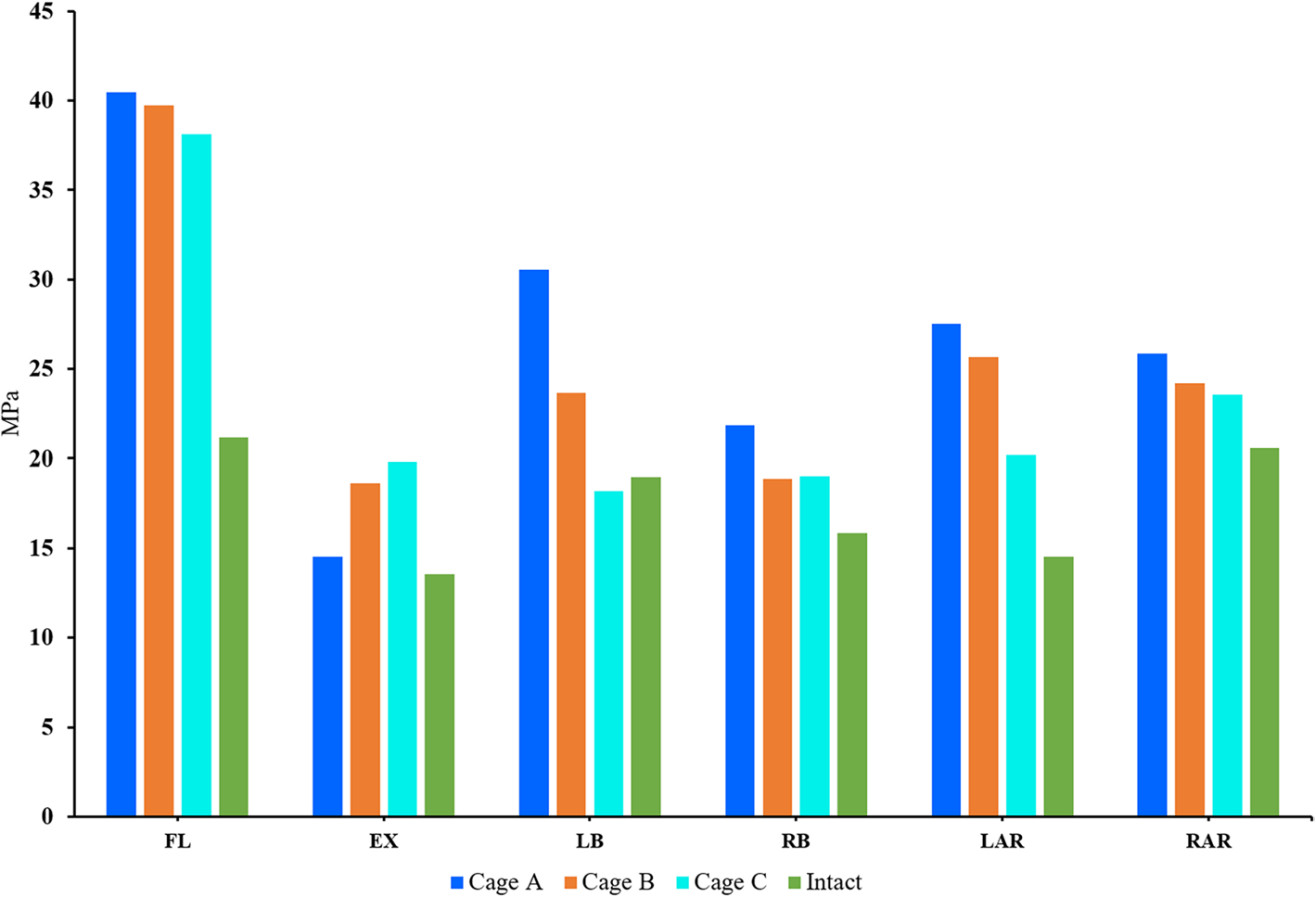
Comparison of the endplate stress between the different FE models

**Fig. 12:**
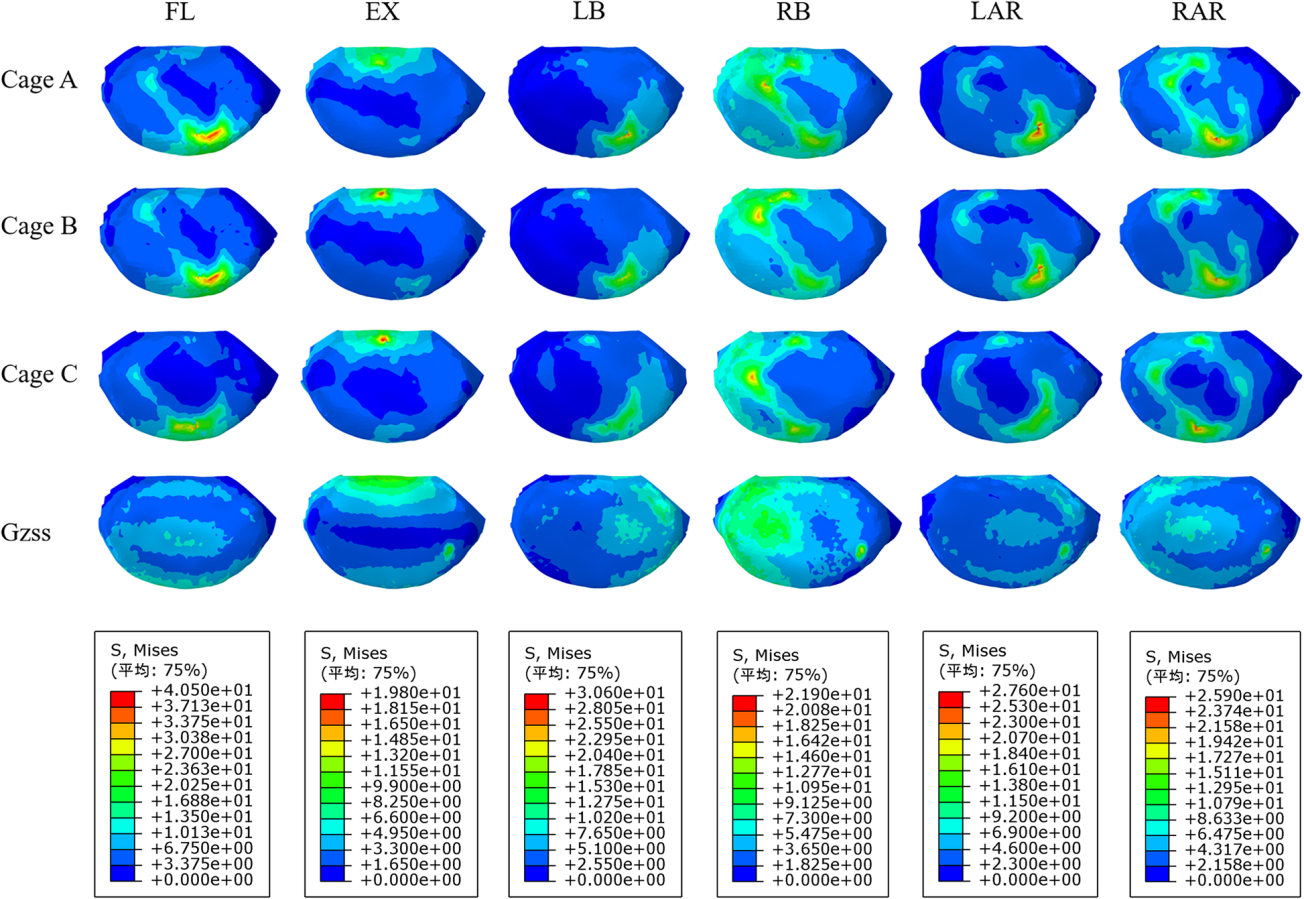
The stress distribution of the endplate at the interface between the cage and L5 upper endplate for the different FE models

The magnitude and trend of stress at the interface between cage and endplate of each model were shown in Figs. 11 and 12. The results showed that the endplate stress of postoperative models was larger than that of the intact osteoporosis model in all directions. On the whole, except for the extension, the stress of the model A-C endplate changed from large to small in other directions. And the difference of endplate stress between postoperative models was the largest during lateral bending. The endplate stress of Model A and model B was 150.5% and 140.9% of that of model C, respectively. Compared with other motion directions, the endplate stress of postoperative models was the highest during flexion. the difference of endplate stress between models A-C and the intact osteoporosis model was the largest in flexion, which was 191.2%, 187.6%, and 180.1% of the intact osteoporosis model, respectively.

## Discussion

Osteoporosis as a common disease in the elderly, with the progress of the aging population, the number of osteoporosis patients undergoing interbody fusion is increasing, while the postoperative complications such as bone non-union and cage subsidence have attracted wide attention because of their serious impact on the prognosis of the patients. How to reduce the postoperative complications of interbody fusion is still a major challenge for surgeons [4]. Therefore, it requires surgeons to improve their surgical techniques, develop better surgical instruments, and more ideal biological agents. Although previous in vitro experiments have studied the mechanical characteristics of different types of cage in spine with different BMD [18–21]. However, there is a lack of specific biomechanical studies on the effect of bone-cage-bone interface area on cage subsidence and other structures of the spine. In this study, FE analysis was used to evaluate the biomechanical characteristics of each tissue structure of osteoporosis model undergoing interbody fusion with different types of cage.

Whether the bone union after interbody fusion can be judged by the follow-up data and clinical symptoms, but limited by the characteristics of FE analysis, we can not judge whether the graft bone has been successfully fused with upper and lower endplate through clinical experience. According to FDA, the criteria for successful interbody fusion are as follow: the translation motion less than 3mmand angular motion less than 5° [55]. The results of this study showed that the ROM of postoperative models was significantly lower than that of the intact osteoporosis model in the L4/5 fusion segment, and had good stability in all directions. The bone-cage-bone interface area of model B and model C was 113% and 167% of that of model A, respectively. The difference was that cage B was increased in length, while cage C was increased in width. With the increase of bone-cage-bone interface area, the postoperative models showed a decreasing trend of ROM at the fusion segment. The larger interface area means that more bone grafts can contact with the vertebral body. This promotes bone union, stabilizes the fusion segment and optimizes the load conduction pathway which is consistent with previous research results [19, 56, 57]. As reported by Jones et al., stand-alone interbody fusion may additionally increase the risk of cage subsidence, especially when osteoporotic, the risk may increase to 2.5 times [58]. The decrease of BMD in osteoporotic people results in a reduction in the overall stiffness of the spine, thus allowing greater mobility of the spinal segments. Good instrument fixation can stabilize the surgical segment well, thereby reducing the probability of cage subsidence.

Rigid fixation of the motion segments may cause the loss of normal activity, resulting in the compensatory increase of the ROM and IDP of the adjacent segment, thus accelerating degeneration and increasing the risk of ASD [59, 60]. ASD can cause severe back pain, radicular symptoms, or neurogenic intermittent claudication that can seriously affect People's Daily lives. It has been reported that the second operation rate of adjacent segment disease is 4% per year, 16.5% in 5 years, and up to 36.1% in 10 years [61]. Therefore, the mechanical changes of adjacent segments are also the focus of this study. Previous studies on the pathogenic factors of ASD have found that decompression of non-fusion segments, the level of fusion segments and the degree of degeneration of adjacent segments highly affect the occurrence of ASD, while the surgical approach and the use of instruments including interbody fusion devices and pedicle screws do not increase the incidence of ASD [62, 63]. In this study, although the ROM and IDP in the cephalic adjacent segment of postoperative models were higher than those of the intact osteoporosis model, there was no significant difference between postoperative models, which was consistent with the previous research results. Although cage and additional posterior instrumentation limit the movement of fusion segment, allowing a compensatory increase in motion in adjacent segments and accelerating the development of ASD. However, the results of this study showed that there was no significant difference in promoting the development of ASD between different axial areas of cage. In addition, the 3D printed cage used in this study can achieve ideal geometric contact between cage and vertebral body. Whether the length or width of cage is prolonged, the increased bone-cage-bone area provides better stability without increasing the risk of ASD.

As a long-term postoperative complication, the cage subsidence reduces the intervertebral space height of the fusion segment to a certain extent, weakens the support of the anterior column, and increases the load-bearing pressure of the posterior approach, which leads to recurrent low back pain and nervous system symptoms, failure of internal fixation and increase of reoperation rate [10, 64]. The stress of cage-endplate interface is the main factor leading to cage subsidence [11]. Previous studies [19, 65, 66] supported the placement of cage on the vertebral epiphysis for interbody fusion, which can not only provide immediate postoperative stability, but also better reduce the incidence of cage subsidence. This is also confirmed by the results of the present study, where endplate stress decreases as the axial area of the cage increases. The endplate has the characteristics of anisotropy. Compared with the central part of the endplate, the peripheral epiphysis is harder and more supportive [67]. Although the biomechanical properties of the endplate were not simulated in this study, it also reflected that a larger axial area of cage can better disperse the pressure borne by the endplate, thereby reducing the risk of cage subsidence, which was consistent with the previous research results [20]. Although the highest endplate stress (40.5 MPa) of the postoperative models was much lower than that of the destructive strength of normal cortical bone (90-200 MPa) [50], this experimental study was an osteoporotic model, and the decrease of BMD will also reduce the cortical stiffness to a certain extent, so this does not mean that it will not lead to endplate damage. And it has been reported previously that the increase of BMI may be associated with the increased risk of cage subsidence after interbody fusion [68, 69]. Our analysis is based on a specific condition, and the real spinal motion is more complex and does not represent the mechanical characteristics of people with different BMI. However, our results reflected the trend that a larger axial area of cage resulted in lower endplate stress in postoperative osteoporosis models.

Previous studies [47] have indicated that that the application of interbody fusion cage and screw-rod system establishes an effective stress conduction pathway, and the use of cage can bear more pressure in the anterior column, so that the stress of posterior instrumentation can be dispersed. Our research results showed that the screw rod stress of the model A-C tended to become smaller. With the increase of the bone-cage-bone interface area, the bearing capacity of the vertebral body is enhanced, and the stress of the internal fixation system is better dispersed. In this study, the maximum posterior instrumentation stress (102.0 MPa) of postoperative models was much less than the yield strength of titanium (825-895 MPa) [50], which was within the safe range. However, a as mentioned above, the FE study only analyzes the mechanical characteristics under specific conditions, which is different from the complex movements in daily life. However, the increasing trend of screw rod stress will bring more risks to the future screw rod failure. such as screw rod loosening, fracture and so on.

There are some limitations in our research. First of all, the intervertebral disc and ligament were simplified in this study, and the material was defined as isotropy, which could not reflect the changes of human motion more accurately. Secondly, the model in this study derived from a healthy adult male, and was not statistically analysed, which is a common defect of finite element analysis. In addition, the stress of adjacent facet joints was not calculated and analyzed in this study, and the risk of ASD was analyzed only in terms of ROM and IDP. The degeneration of articular process also plays an important role in the occurrence of adjacent spondylosis. Therefore, the risk analysis of ASD is not comprehensive enough. The results of the analysis were carried out under specific conditions, and more represent the overall trend, and the conclusions should be combined with in vitro experiments. It is a pity that this study did not carry out a more detailed division of osteoporotic population, and there was no further comparative analysis of the mechanical changes of different axial area of cage under different BMD, which is a pity for us. In the next step, we will improve our experimental methods and conduct more reasonable research.

## Conclusions

In osteoporosis patients undergoing interbody fusion, the use of larger cage sizes, whether increased in length or width, will provide better immediate stability in all directions of motion. A larger cage means a larger contact area. This personalized cage can make more grafts come into contact with the vertebral body, and the porous part of 3D printing cage can be better induced and fused with the vertebral body. In addition, the application of different types of cage does not cause significant changes in the ROM and IDP of the proximal cephalic segment, and does not increase the possibility of the occurrence of ASD. The larger cage optimizes the load transmission pathway of the spine, and its larger contact area can better disperse the stress borne by the endplate, reducing the occurrence of stress concentration. In addition, a larger cage can be placed in more contact with the surrounding endplate, so that the more rigid epiphysis can play a role, reducing the phenomenon that only the central endplate bears stress, which has a positive significance in preventing endplate fracture and cage subsidence. The application of cage and the screw rod system established an effective stress conduction pathway in the spine. The rigid fixation of the screw-rod system stabilizes the surgical segment, reduces the ROM of the fixed segment. The pedicle screw can better support the anterior column and reduce the pressure of cage. On the other hand, the larger cage greatly enhances the stress bearing capacity of the front column, can better distribute the stress of the posterior spine structure and the stress borne by the posterior screw rod system, reducing the stress concentration of the screw rod system, which exceeds the yield strength of the material, resulting in the risk of future instrument failure.

## Data Availability

The datasets used and/or analysed during the current study available from the corresponding author on reasonable request.

## Abbreviations

FE: Finite element
ROM: Ranges of motion
IDP: Intradiscal pressure
ASD: Adjacent segment disease
PLIF: Posterior lumbar interbody fusion
LLIF: Lateral lumbar interbody fusion
TLIF: Transforminal lumbar interbody fusion
